# Viewpoint on Human Immunodeficiency Virus Medical Care Retention Guidelines in the Coronavirus 2019 Pandemic Era and Beyond: Lessons Learned From Electronic Health Record Screening and Outreach

**DOI:** 10.1093/ofid/ofae031

**Published:** 2024-01-16

**Authors:** Ethan Moitra, Paola C Jiménez Muñoz, Martha C Sanchez, Megan M Pinkston

**Affiliations:** Department of Psychiatry and Human Behavior, Alpert Medical School of Brown University, Providence, Rhode Island, USA; Mailman School of Public Health, Columbia University, New York, New York, USA; Infectious Diseases and Immunology, The Miriam Hospital, Providence, Rhode Island, USA; Department of Medicine, Alpert Medical School of Brown University, Providence, Rhode Island, USA; Department of Psychiatry and Human Behavior, Alpert Medical School of Brown University, Providence, Rhode Island, USA; Department of Medicine, Alpert Medical School of Brown University, Providence, Rhode Island, USA; Lifespan Physicians Group, Rhode Island Hospital, Providence, Rhode Island, USA

**Keywords:** electronic health record, HIV, retention, telehealth

## Abstract

In this viewpoint, we discuss retention in care for people with human immunodeficiency virus (HIV) and call into question the methodology used to characterize retention, as well as the definitions themselves. Optimal retention for people with HIV (PWH) is defined in multiple ways by major healthcare leaders in the United States, typically focusing on appointment attendance or laboratory work. Yet, these definitions rely on in-person encounters, an approach to care that is becoming less common due to the rise of telehealth visits, particularly in light of the coronavirus disease 2019 pandemic. Our recent work showed that relying on electronic health records to identify PWH who were not retained in care not only failed to capture the nuances of modern HIV medical treatment engagement, but also led to misidentification of patients’ retention status due to limitations in the record system. As such, we recommend a reevaluation of how HIV medical care retention is defined and reported.

## Teaser

A rethinking of HIV care retention definitions is recommended to account for real-world practice. Reliance on electronic health record systems to identify out-of-care people with HIV has limitations.

In 2021, 46.1% of individuals living with human immunodeficiency virus (HIV) in the United States (US) were classified as not regularly retained in medical care [1]. People with HIV (PWH) who do not engage in consistent, ongoing medical services risk a number of critical consequences to their health and quality of life, and are at increased risk of transmitting HIV [2, 3]. Most notably, patients who have difficulty engaging in care have a reduced likelihood of timely initiation of antiretroviral therapy (ART) [4]. Once ART is initiated, patients who are not retained in care are also less likely to take medications as prescribed, increasing the risk of viral drug resistance [5] and HIV transmission [6]. Many PWH in the US fail to achieve sustained viral suppression, in part because of gaps in their engagement and retention in care [7]. Overall, poorly retained PWH are likely responsible for more HIV transmissions than the population in care and the undiagnosed population combined [2, 8]. In sum, retention in HIV care is essential for the long-term health and well-being of PWH and is of great public health concern. However, as discussed in this viewpoint, the methodology used to characterize retention, as well as to define it, has limitations.

## DEFINING HIV MEDICAL CARE RETENTION

Retention in HIV care has been defined in a variety of ways, with definitions falling under either laboratory-based or appointment-based criteria. Historically, retention in care correlated with viral suppression and survival. Indeed, it was essential in the early era of HIV treatment when ART was less effective and potentially toxic. According to the Centers for Disease Control and Prevention (CDC), retention is defined as PWH completing ≥2 CD4 or viral load (HIV RNA) tests at least 3 months apart in 12 months [1]. The National HIV/AIDS strategy uses the same definition [9]. The US Health Resources and Services Administration (HRSA) defines retention as PWH attending at least 2 medical visits that were at least 90 days apart in 12 months [10]. This aligns with the Institute of Medicine's (IOM) definition of retention [11]. A commonality across these metrics is that the encounters (ie, laboratory work, appointments) occur in-person.

Yet, recent research findings suggest that previous reports overestimated the proportion of PWH who were defined as “out-of-care” based on these national measures. For instance, across 3 different studies, 72%–79% of PWH who were characterized as out-of-care had either moved, had engaged in care elsewhere, had died, were incarcerated, or were otherwise mistakenly placed in this category [12–15]. These significant error rates reflect the need for better measures of retention in care [16].

## HIV CARE IN THE COVID-19 PANDEMIC ERA

The coronavirus disease 2019 (COVID-19) pandemic led to widespread lockdowns to mitigate the spread of the severe acute respiratory syndrome coronavirus 2. In the medical community, many nonemergent outpatient clinical services were limited or suspended, virology laboratories closed or de-prioritized non-COVID-19 activities, and physical spaces were reconfigured to reduce transmission risk or to account for the treatment of patients with COVID-19 [17, 18]. Additionally, the use of telehealth services proliferated during the pandemic [19]. Although the World Health Organization declared an end to COVID-19 as a public health emergency in May 2023, approaches to medical treatment that expanded during the pandemic persist.

In the HIV care landscape, numerous services changed, reduced, or outright stopped. For example, our group's work in geographically diverse HIV testing sites showed substantial decreases in testing early in the pandemic [20]. Results showed a rebound in most testing services, although to our knowledge, some services have yet to return to prepandemic levels (eg, mobile HIV testing). In terms of outpatient HIV medical services, data also illustrate the expansion of telehealth-based care or other virtual care options [21]. However, remote services for PWH are not new. In particular, they have been tested among hard-to-reach populations or in situations where provider resources are limited, such as in low- and middle-income countries [22]. In a prepandemic study of PWH in 2018, 57% of participants expressed openness to using telehealth for their HIV care and 37% would be willing to use telehealth frequently or always as an alternative to clinic visits [23]. During the pandemic, similar results were found in terms of patients being equally or more likely to attend appointments if they were offered remotely [24] and that telehealth visits were acceptable to most PWH [25]. Moreover, data show that telehealth visits are associated with improved viral load suppression [26] and overall appointment attendance [24, 27]. Finally, a recent systematic review and meta-analysis demonstrated that telehealth visits for PWH are associated with increased ART adherence [28]. Prior to the pandemic, telehealth visits in the US were rare and usually not covered by insurance carriers. However, in the pandemic and postpandemic era, service delivery and payor coverage have expanded. We suggest that defining retention based on in-person services alone loses sight of the evolving approach to HIV care in the US, which was particularly accelerated by the COVID-19 pandemic.

Irrespective of the pandemic, the potential benefits of telehealth services for PWH are noteworthy, including decreasing travel time to clinics, affording flexible scheduling, and mitigating stigmatization fears because patients do not need to physically enter an HIV care site. However, it is important to acknowledge the limitations of telehealth, particularly among individuals already challenged by social determinants of health. For example, recent studies show that technological challenges, limited access to technology, and low digital literacy can limit equitable access to and benefits from telehealth for HIV [29, 30]. Data also show that PWH who self-identify as people of color, a group that has historically experienced health inequities contributing to lower rates of treatment retention [31], can be less likely to use telehealth services [32]. Clearly, telehealth services have limitations and might not be ideal for some patients. Nevertheless, in this viewpoint, we join a growing group of clinicians and researchers who acknowledge that telehealth services are here to stay. Furthermore, consistent with other commentaries [25], telehealth services need to be considered when defining HIV care retention.

## FINDINGS FROM THE ARC STUDY

Building on prior research and viewpoints, we share findings from an ongoing study to illustrate how extant definitions of retention do not reflect current HIV care practices. We are currently conducting a pilot project called the Acceptance and Reduced Stigma to Improve Retention in Care (ARC) study. In this project, we aim to recruit English- and Spanish-speaking PWH who use substances (ie, cocaine, methamphetamine, opioids) and who are poorly retained in care. Initially, our recruitment site, an outpatient infectious diseases (ID) clinic in Providence, Rhode Island, which provides care to nearly 2000 patients, generated a list of 434 potentially eligible patients using prespecified parameters through an electronic health record (EHR) data screen. Alarmingly, more than half of the patients (n = 230/434 [53%]) had not attended an in-person clinic appointment in ≥9 months.

To confirm likely eligibility prior to outreach, study staff then conducted an in-depth EHR review of each patient on the list. Further scrutinization revealed that 36 patients transferred HIV care due to moving out of the state or country; another 10 patients were suspected to have moved out of the state or country. Four patients still living in Rhode Island were confirmed to have transferred HIV care elsewhere, and 5 patients were incarcerated. Unfortunately, our review also revealed that 7 patients were deceased.

Among our remaining eligible sample, we discovered that 86 patients had at least 1 non-HIV-related medical encounter in the local healthcare system (eg, primary care or urgent care/emergency department visit) during their period of being out of care and/or missing appointments at the ID clinic. Moreover, 150 patients received ART refills despite not being seen in the clinic and/or missing appointments for ≥6 months. Last, 13 patients appeared for 1 or more telehealth encounters with a medical provider within a ≥6 month out-of-care period in which they missed 1 or more in-person appointments. Thus, many of these patients were evidencing signs of care engagement. However, these indices would not be captured in the traditional definitions of retention. In sum, after starting with a list 434 PWH suspected to be out of care, we found that 14.3% (n = 62) were no longer patients at the site (eg, due to moving, transfer of care, death) and that 57.4% (n = 249) showed some evidence of engagement with the ID clinic or the wider medical system.

## CONCLUSIONS AND RECOMMENDATIONS

In light of our findings, we emphasize the importance of reconsidering how HIV medical care retention is defined and reported. Our results add to the burgeoning area of work [12–15] that has demonstrated that current measures of retention in care can lead to a high error rate, resulting in an overestimation of individuals who are out of care. Accumulating data indicate the need for a closer data review to measure variables (eg, migration, deaths, data entry mistakes, treated elsewhere) that are contributing to these errors because solely relying on surveillance data is not likely to yield valid results. Indeed, inaccurate retention in care metrics could impact resource allocation by sending funds to sites that have mistakenly overestimated the number of out-of-care patients in their panel. Instead of using these funds to attempt to track down patients who have moved out of the area or might be deceased, these funds could be redirected to clinics with reliable data and valid patient needs.

Additional measures to quantify retention in HIV healthcare would be prudent to establish, not only to improve characterization of out-of-care patients, but also to standardize monitoring across HIV care clinics. Our experience working in an ID clinic suggests that providers have characterized patients as being retained in care through other metrics. These include gaps in care (ie, how long since the patient completed a medical visit), appointment attendance (ie, how many scheduled appointments were kept), and results of laboratory investigations (ie, whether the patient has a detectable viral load). Additionally, our results reflect that a high number of individuals who met clinic criteria for being out of care received refills for ART without provider contact. Given that our study is one of a number of studies reflecting overestimation of out-of-care statistics, we recommend that attention be placed on revising retention measurement algorithms (eg, HRSA performance measures), such as those receiving Ryan White federal funding, to ensure accurate representation of their retention rates. With accurate data, resources can be redirected toward patients who are truly out of care and who likely face greater behavioral, medical, and social needs that prevent their engagement in care.

Since COVID-19, the necessity for and the frequency of telehealth visits have increased, yet telehealth visits in HIV care are not included in measures of retention. We recommend that attended telehealth visits count toward retention. Consistent with retention criteria from HRSA and IOM, ≥2 telehealth visits (or a combination of in-person and telehealth) should be completed at least 90 days apart in 12 months. If telehealth visits were to be included as a measure of retention, important questions should be considered: Who is offered telehealth? Should it vary by patient characteristics, such as being newly diagnosed or having uncontrolled viral load? Will receipt of telehealth affect engagement in other services? Does a telehealth visit substitute for an in-person appointment? If so, how often?

Regarding the CDC and National HIV/AIDS Strategy guidelines that define retention according to CD4 or viral load tests, we recommend extending the window of testing to ≥2 CD4 or viral load tests at least 6 months apart in 12–24 months. We suggest this will better reflect real-world practice, particularly for medically stable patients. On a related note, attention is needed to determine how use of remote prescription refills fits into retention definitions. For instance, if a patient has stable laboratory values and engages in the behavior of obtaining their medications, this suggests they are engaging in behaviors consistent with HIV management. Additionally, if a patient is stable and receives the refill from their HIV care provider, one may question the purpose of attending a medical appointment. We recommend that prescription refill pickup should count toward retention, but only if there is an attended in-person or telehealth visit within 12 months of the refill. Overall, these recommendations can serve to expand upon existing US HIV care retention guidelines (see Table 1 for summary of recommendations).

**Table 1.** Recommended Revisions to Human Immunodeficiency Virus Retention in Care Measures in the United States

Current Guidelines	Recommended Revisions
CDC and National HIV/AIDS strategy: ≥2 CD4 or viral load tests at least 3 mo apart in 12 mo	≥2 CD4 or viral load tests at least 6 mo apart in 12–24 mo
HRSA & IOM: ≥2 medical visits at least 90 d apart in 12 mo	≥2 telehealth visits (or combination of in-person and telehealth) at least 90 d apart in 12 mo
None regarding prescription refills	Remote prescription refill pickup, only if there is an attended in-person or telehealth visit within 12 mo of the refill

Abbreviations: CDC, Centers for Disease Control and Prevention; HRSA, US Health Resources and Services Administration; IOM, Institute of Medicine.

Next, our data suggest that although individuals were counted as out of care for HIV, it was frequently observed in medical charts that individuals were engaged in other healthcare services. For instance, there were patients who were “out of care” yet had communications with clinic staff (eg, calls by staff to update Ryan White forms, patient calls to review laboratory results or ask for case management support needs), sometimes multiple times, but the patients were not scheduled for appointments or asked about their lack of engagement in care. The use of EHRs could improve upon retention in care measurement by measuring not only HIV-specific care, but also other specialized and holistic healthcare that individuals are receiving. Data suggest that ancillary care services for PWH, such as non-HIV medical care, case management, behavioral health services, and housing assistance [33], might constitute an indirect, but promising pathway to connecting with out-of-care PWH to reengage them in HIV services [34]. We recommend that out-of-care PWH be flagged in EHR systems to prompt staff at any clinic within the local healthcare system to inquire about HIV treatment engagement, encourage appointment scheduling/attendance, and otherwise connect the patient to the HIV provider's office. This is consistent with the recommendation to leverage HIV “status-neutral” approaches to destigmatize HIV and make care more approachable [35]. Services that are more holistic and include aspects of wraparound approaches might be better suited to meet the needs of PWH, as well as overcome stigma-related barriers.

On an aspirational level, it is promising and important to acknowledge that the overestimation of PWH out of care also suggests that the US might be closer to achieving retention results in line with the Joint United Nations Programme on HIV/AIDS 95–95-95 goals for HIV in the US [36]. than is currently believed. This viewpoint is focused on HIV care in the US. However, we suggest that many of the lessons learned and recommendations can translate to other countries. By enhancing system enablers and generating more accurate data, resources may be better directed to the individuals in greatest need and reduce HIV-related disparities and health inequities. Furthermore, the acknowledgement of engagement in healthcare services outside of specific HIV care is important to understand given continued HIV stigma and the potential benefits of status-neutral care.

## Notes

***Acknowledgments.*** E. M. and M. M. P. conceptualized and designed the study. P. C. J. M. led all data-related activities. E. M. wrote the first draft. All authors contributed to the literature review and discussion.

***Patient consent.*** This work did not necessitate patient consent. However, a waiver of consent was obtained to review electronic health records as part of our parent project. This waiver was approved by the Lifespan/Rhode Island Hospital Institutional Review Board 3 (number 1822922-5).

***Financial support.*** This work was supported by the Providence/Boston Center for AIDS Research (National Institute of Allergy and Infectious Diseases, grant number P30 AI042853) and the National Institute on Drug Abuse (grant number R34 DA053738 to E. M. and M. M. P.).
